# Mesotrione herbicide promotes biochemical changes and DNA damage in two fish species

**DOI:** 10.1016/j.toxrep.2015.08.007

**Published:** 2015-08-22

**Authors:** L.D.S. Piancini, I.C. Guiloski, H.C. Silva de Assis, M.M. Cestari

**Affiliations:** a Universidade Federal do Paraná, Departamento de Genética, Curitiba, Paraná, Brazil; b Universidade Federal do Paraná, Departamento de Farmacologia, Curitiba, Paraná, Brazil

**Keywords:** Mesotrione, Herbicide, Genotoxicity, Fish, Neotropical, ROS

## Abstract

Mesotrione is one of the new herbicides that have emerged as an alternative after the ban of atrazine in the European Union. To our knowledge, any work using genetic or biochemical biomarkers was performed in any kind of fish evaluating the toxicity of this compound. The impact of acute (96 h) exposure to environmentally relevant mesotrione concentrations (1.8, 7, 30, 115 e 460 μg L^−1^) were evaluated on the liver of *Oreochorimis niloticus* and *Geophagus brasiliensis* by assessing the activity of superoxide dismutase (SOD), glutathione peroxidase (GPx) and glutathione-S- transferase (GST), the levels of reduced glutathione (GSH), carbonyl assays (PCO) and lipid peroxide (LPO) as well as the DNA damage to erithrocytes, liver and gills through the comet assay. We observed an increase in the concentration of GSH and the GPx activity in *O. niloticus*, and the GST and SOD activity in *G. brasiliensis*. We found significant increase in DNA damage in all tissues in both species. The results indicated that the acute exposure to mesotrione can induce oxidative stress and DNA damage in both species.

## Highlights

• Our results are novel, never before the toxicity of mesotrione was tested with biomarkers in fish
• We assess the acute effects of mesotrione using 9 different biomarkers.
• DNA damage was assessed in three different cell types in two species exposed to mesotrione.
• We found a positive ROS response in *O. niloticus* and *G. brasiliensis* induced by mesotrione.
• Adverse effects were found on concentrations lower than the applied on crop fields.

## 1 Introduction

Herbicides can pollute nearby aquatic ecosystems through spray drift, soil leaching, or runoff, potentially leading to effects on nontarget species. Therefore, the contamination of aquatic ecosystems by pesticide is therefore an environmental concern [6].

The banishment of agrochemicals in many countries, *e.g.*, atrazine in European countries, has driven the development of new selective herbicides. Mesotrione is a selective preemergent and postemergent herbicide that can control a variety of common turfgrass weeds [20]. This herbicide is unstable in the environment, and it is detected in soil and water just a short time after application [5].

Nevertheless, the metabolite MNBA (4-methylsulfonyl-2-nitrobenzoic acid) is detected with the parent mesotrione in crops, and two degradation products, MNBA and AMBA (2-amino-4-methylsulfonylbenzoic acid), are found together with the parent compound in soil and water [1]. Toxic effects of these mesotrione metabolites has been found by Bonnet et al. [6] in two microorganisms. Crouzet et al. [11] tested the effects of mesotrione over soil microbial community, at doses far exceeding the recommended rates, mesotrione had impacts on these non-target soil microorganisms. Kreutz et al. [23] evaluated the LC_50_ of this compound in the neotropical fish *Rhamdia quelen*. No work testing the potential sub-lethal effects of mesotrione through a biomarkers approach was performed in any species of fish.

The effectiveness of biomarkers has been demonstrated in several studies on the toxicity of pesticides to fish [16], [25], [32]. Under physiological conditions, reactive oxygen species (ROS) are continuously produced by O_2_ metabolism. Antioxidant enzymes such as superoxide dismutase (SOD), catalase (CAT), and glutathione peroxidase (GPx) scavenge these free radicals to prevent oxidative damage. The glutathione-S-transferase (GST) is a ohase II enzyme that mediates the conjugation of xenobiotics with reduced glutathione (GSH) in many organisms, including plants, mammals and fish [38]. ROS are commonly associated with cellular injuries, especially with alterations to macromolecules, such as the DNA, lipids, and proteins. Several tests are known to quantify damage in those molecules, *e.g.*, comet assay for DNA, lipid peroxidation (LPO) for membranes, and protein carbonylation (PCO) for proteins, and thus have high predictive value as effect biomarkers [28].

Fish are often used as sentinel organisms for ecotoxicological studies because they play numerous roles in the trophic web, they can toxic substances and respond to low concentration of xenobiotics [7]. *O*reochorimis* niloticus* and *G*eophagus* brasiliensis* were chosen as bioindicators in this study due its easy adaptation to laboratory conditions, wide distribution and easy to obtain, and they are extensively consumed by humans. Despite being used in the field over 15 years, there are very few studies that evaluate possible adverse effects of mesotrione in living organisms, and any test on fish using biomarkers was performed. Thus, this study aims to evaluate if this compound may generate oxidative stress and DNA damage in these two species.

## 2 Material and methods

Mesotrione (CAS 104206-82-8) concentrations were obtained from a stock solution prepared in distilled water at 136.40 mg L^−1^.

Fish were exposed to mesotrione in the nominate concentrations of 1.8, 7, 30, 115, and 460 μg L^−1^. The concentration of 1.8 μg L^−1^ is an ecologically relevant concentration, since this value is found in runoff in crop fields [5]. The other concentrations were determined as a fourfold of the previous concentration.

### 2.1 Reagents

Normal agarose, Low melting point agarose, *Tris* (hydroxymethylaminomethane), EDTA (ethylene- diamine-tetraacetic acid), β-NADPH (reduced nicotinamide-adenine dinucleotide phosphate), DTNB (5,50-dithiobis(2-nitrobenzoic acid)), BHT (butylated hydroxytoluene), and FeSO_4_NH_4_ (ammonium ferrous sulfate) were acquired from Sigma–Aldrich Corporation (USA). Bovine serum albumin was acquired from Bio-Rad Laboratories (USA), and DMSO (dimethyl sulfoxide) from Merck Corporation (Germany). The herbicide Mesotrione was acquired from Chem Service Inc (USA). All other reagents were from local suppliers (Brazil) and they were of analytical grade.

### 2.2 Experimental design

Specimens of *O. niloticus* and *G. brasiliensis* were acquired from a local fish farm. The fish were acclimatized for 60 days in tanks of 250 L with filtered water, constant aeration, average temperature of 22 °C, photoperiod of 12 h, and daily feeding. Two weeks before the beginning of the experiment, 105 fish were randomly assigned to seven aquaria of 108 L (15 specimens per aquarium) in conditions similar to the tanks (water, aeration, temperature, *etc*.). One aquarium was assigned as a negative control group (NC), one as positive control (PC), exposed for 24 h to 0.5 mg kg^−1^ of methyl methane sulfonate (MMS) via intraperitonial injection, and the other 5 as treatment groups. We made two separate experiments for each species and in any time, either during acclimatization or exposure, fish of different species shared the same environment.

After a 96 h hydric exposure to mesotrione in a static bioassay, fish were anesthetized with benzocaine 10%. Blood was sampled through caudal vein puncture, and then the fish were rapidly killed by spinal cord section. The liver was removed and placed in a petri dish. A sample of the organ was separated with a scalpel and placed in a microtube containing 0.5 mL of fetal bovine serum (FBS, Invitrogen). The other part of the liver was stored at – 80 °C for the biochemical analysis. The third gill arch of the right side of each fish was excised and placed in a petri dish and washed in phosphate buffer solution (PBS, pH 7.4). The bone arch was removed with a scalpel and only the lamellae were transferred to a microtube containing 0.5 mL of FBS.

### 2.3 Comet assay

The comet assay with peripheral blood (erythrocytes; ECA) was performed according to Speit and Hartmann [35], modified by Cestari et al. [9] and Ferraro et al. [14]. For the gill (GCA) and liver (LCA) comet assay, the organs were mechanically homogenized (homogenizer Tecnal-TE-103) at 1500 rpm [31]. Ten microliters *aliquot* was taken from each diluted sample and embedded in 120 μL of low- melting-point agarose (0.5%, Invitrogen). The following steps were conducted according to Speit and Hartmann [35].

One hundred nucleoids were analyzed for each organ and blood in each fish according to the visual classification based on the migration of DNA fragments from the nucleus. The results were categorized into classes according to [10]: class 0 (no visible damage), class 1 (little damage), class 2 (medium damage), class 3 (extensive dam- age) and 4 (maximally damaged). The score was calculated by multiplying the number of nuclei found in a class times the class number.

### 2.4 Biochemical biomarkers

Pools of liver of two individuals were used for the assays of the enzymatic activities of superoxide dismutase (SOD), glutathione peroxidase (GPx) glutathione S-transferases (GST), and for the determination of reduced glutathione concentration (GSH), lipid peroxidation levels (LPO) and protein carbonyl assay (PCO). Samples were homogenized in phosphate buffer (0.1 M) at pH 7.0, and centrifuged at 15.000 × *g* for 30 min, at 4 °C.

SOD activity was assayed according to Gao et al. [15]. 40 μL of sample, 885 μL of buffer (1 M Tris-base / 5 mM EDTA, pH 8.0) and 50 μL of pyrogallol (15 mM) were added to a microtube and the solution was incubated for 30 min. The reaction was stopped with 25 μL HCl (1N). In a microplate 200 μL of the solution was added per well, and the absorbance was measured at 440 nm.

GPx activity was measured according to Paglia and Valentine [29]. In microplate 10 μL of sample and 130 μL of reaction medium (3.08 mM of sodium azide; 0.308 mM β-NADPH, reduced nicotinamide-adenine dinucleotide phosphate; 1.54 U/mL glutathione reductase and 3.08 mM reduced glutathione in 0.1 M sodium phosphate buffer, pH 7.0) were added. After two minutes, 60 μL of hydrogen peroxide (1.5 mM) was added. Absorbance was monitored at 340 nm.

GSH was measured according to Sedlak and Lindsay [34]. A volume of 50 μL of supernatant (after protein precipitation by 10% trichloroacetic acid) and 230 μL of TRIS buffer (0.4 M, pH 8.9) were placed in a microplate, followed by addition of 20 μL of 2.5 mM DTNB in 25% methanol. Absorbance was determined at 415 nm.

GST activity was measured using reduced glutathione (GSH- 3 mM) and 1-chloro-2,4-dinitrobenzene (CDNB- 3 mM) as substrates [21]. The absorbance increase was measured at 340 nm.

LPO analysis was carried out using the ferrous oxidation-xylenol assay [18]. A volume of 100 μL of sample were mixed with 900 μL of reaction solution (0.1 mM xylenol orange, 25 mM H_2_SO_4_, 4.0 mM BHT (butylated hydroxytoluene) and 0.25 mM FeSO_4_NH_4_ (ammonium ferrous sulfate) added in this specific order in 90% grade methanol). After 30 min the absorbance was measured at 570 nm.

PCO analysis was conducted at 360 nm by derivatization of the protein carbonyl groups with 2, 4-dinitrophenol hydrazine (10 mM of 2,4-dinitrophenylhydrazine in 2.0 M of hydrochloric acid) to yield dinitrophenyl hydrazones [24].

The protein concentration was determined using Bradford's method (1976), with bovine serum albumin as the standard at 595 nm.

### 2.5 Statistical analysis

Since data obtained from the comet assay analysis is categorical, a non-parametric statistical approach was chosen. The Kruskal–Wallis test was used to compare controls and contaminated treatments for each organ separately. Differences were analyzed by a *post hoc* Student–Newman–Keuls and statistical significance was considered for *p* < 0.05.

Oxidative stress biomarkers were tested for normal distribution using the Kolmogorov–Smirnov test. Parametric one-way analysis of variance (ANOVA) or the non-parametric Kruskal–Wallis test was applied according to data distribution (normality and homogeneity of variance). Differences were analyzed by a *post hoc* Tukey test (after ANOVA) or Student–Newman–Keuls test (after Kruskal–Wallis). Statistical significance was considered for *p* < 0.05.

## 3 Results

There was no animal death in the 96 h of exposure to mesotrione.

### 3.1 *Oreochromis niloticus*

**Fig. 1:**
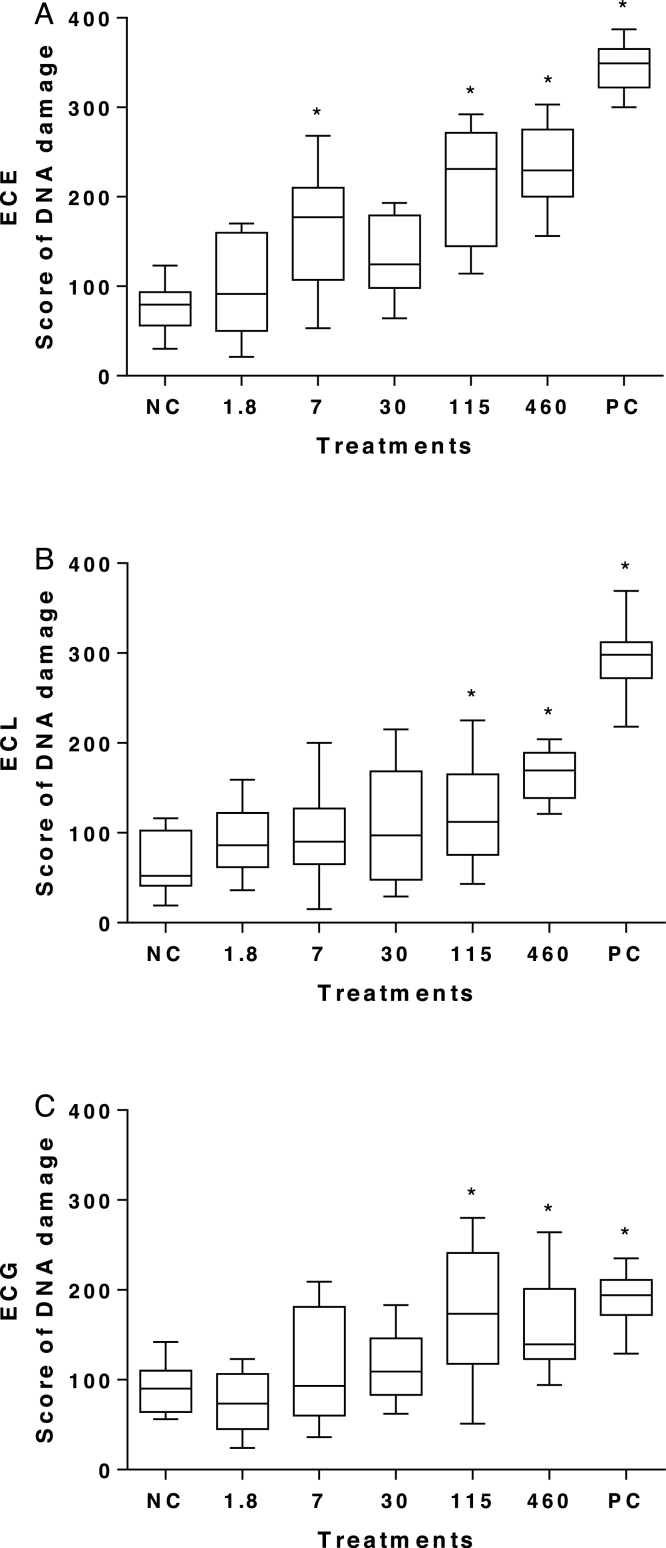
Scores of the comet assay in (A) erythrocytes, (B) hepatocytes, and (C) gills cells of *O. niloticus* exposed to mesotrione. NC: negative control; PC: positive control. Data are present in median and min-max. * represents statistical significance of treatments in comparison to the NC using Kruskal–Wallis test; *p* < 0.05.

Concerning the erythrocytes comet assay (ECA), specimens exposed to 7, 115 e 460 μg L^−1^ showed higher DNA damage in comparison to the NC (Fig. 1A). The gill comet assay (GCA, Fig. 1B) and liver comet assay (LCA, Fig. 1C) presented higher DNA damage in the highest concentrations, 115 e 460 μg L^−1^ of mesotrione.

**Fig. 2:**
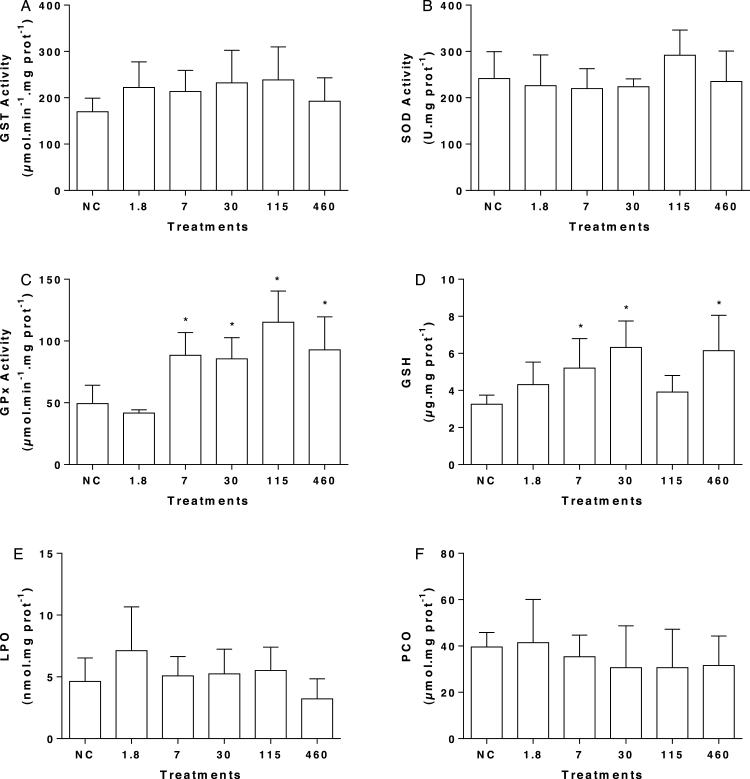
Biochemical biomarkers in *O. niloticus* exposed to mesotrione. (A) GST specific activity, (B) SOD specific activity, (C) GPx specific activity, (D) GSH concentration, (E) lipoperoxidation, and (F) protein carbonylation. Data are present by mean and standard deviation. NC: negative control; PC: positive control. *represents statistical significance of treatments in comparison to the NC using ANOVA; *p* < 0.05.

Acute exposure to mesotrione did not change the activities of GST (Fig. 2A) and SOD activities significantly (Fig. 2B). However, after exposure to 7, 30, 115 e 460 μg L^−1^ of mesotrione the activity of GPx was significantly increased (Fig. 2C). The reduced glutathione content (GSH) was higher in the treatments 7, 30 e 460 μg L^−1^ (Fig. 2D). There was no change in LPO (Fig. 2E) and PCO (Fig 2F) levels in the liver of *O. niloticus*.

### 3.2* G. brasiliensis*

**Fig. 3:**
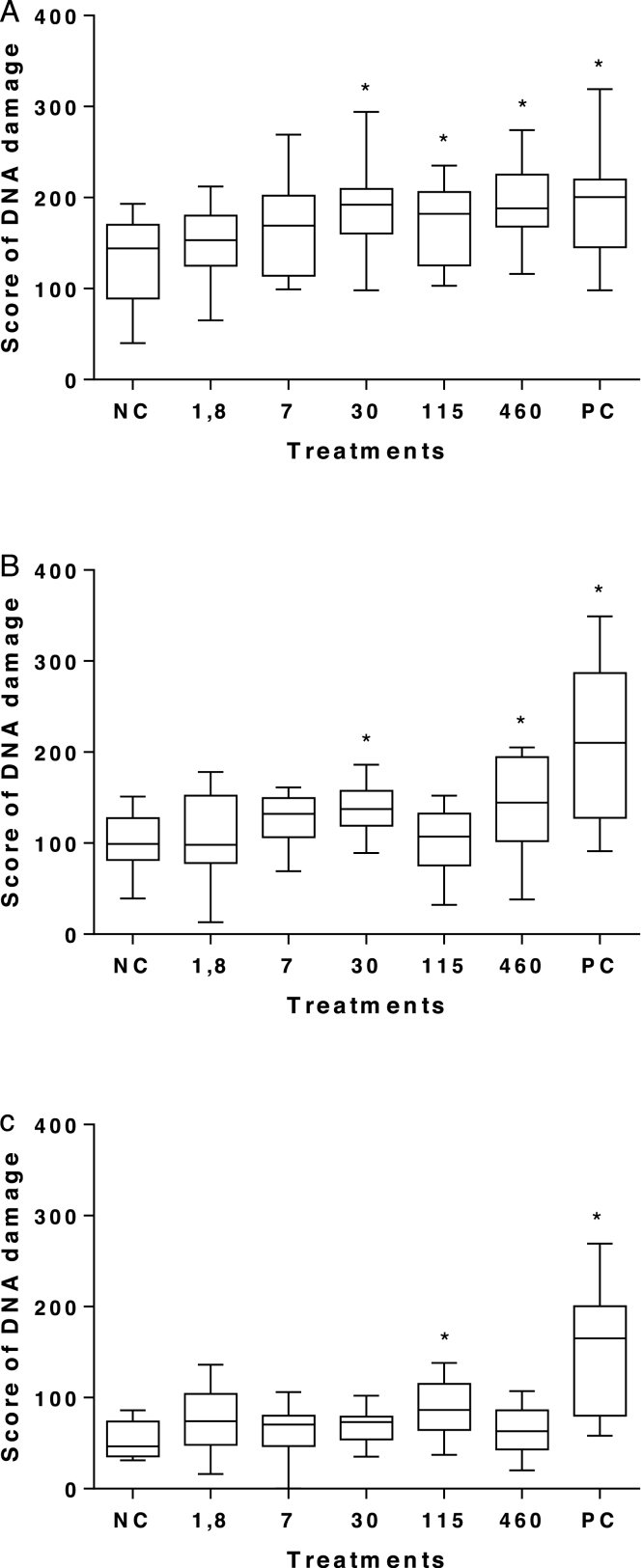
Scores of the comet assay in (A) erythrocytes, (B) hepatocytes, and (C) gills cells of *G. brasiliensis* exposed to mesotrione. Data are present in median and min-max. NC: negative control; PC: positive control. *represents statistical significance of treatments in comparison to the NC using Kruskal–Wallis test; *p* < 0.05.

Mesotrione induced DNA breakages in erythrocytes of the fish exposed to 30, 115 and 460 μg L^−1^ (Fig. 3A). The hepatocytes showed higher DNA damage in 30 and 460 μg L^−1^ (Fig. 3B), and the gill cells only had DNA damage in the 115 μg L^−1^ treatment (Fig. 3C).

**Fig. 4:**
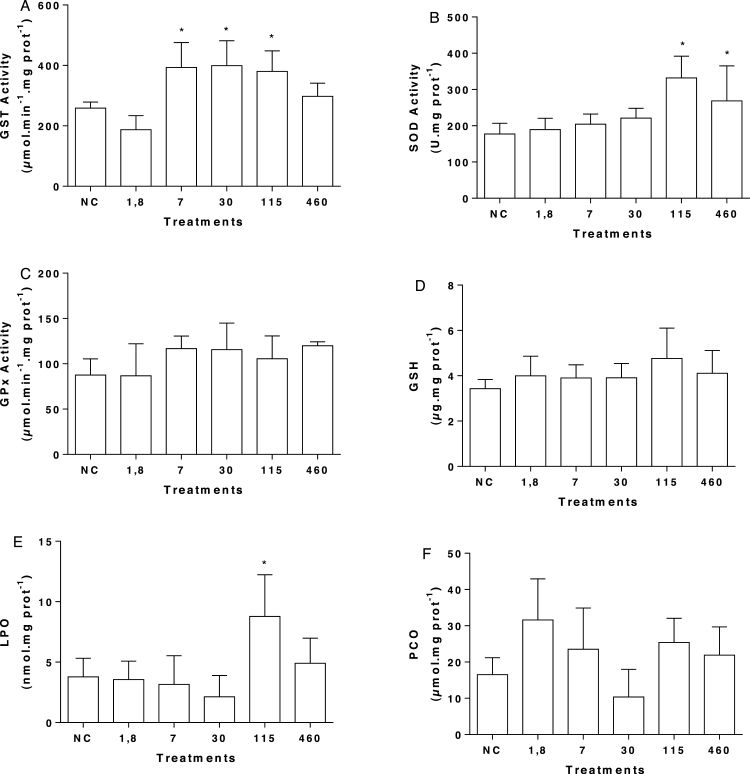
Biochemical biomarkers in *G. brasiliensis* exposed to mesotrione. (A) GST specific activity, (B) SOD specific activity, (C) GPx specific activity, (D) GSH concentration, (E) lipoperoxidation, and (F) protein carbonylation. Data are present by mean and standard deviation. NC: Negative control; PC: positive control. *represents statistical significance of treatments in comparison to the NC using ANOVA; *p* < 0.05.

A meaningful increase in GST activity was found in fish exposed to mesotrione at 7, 30, and 115 μg L^−1^ (Fig. 4A) as well an increase in SOD at 115 and 460 μg L^−1^ treatments (Fig. 4B). We did not observed significantly changes in the GPx activity (Fig. 4C). There was no change in the GSH content in any group (Fig. 4D), but the LPO levels were higher in the 115 μg L^−1^ group. An apparent increase in the PCO levels was observed in fish exposed to 1.8; 7; 115 e 460 μg L^−1^ of mesotrione, but no significance differences were found (Fig. 4F).

## 4 Discussion

Mesotrione is easily and quickly degraded and eluted from the soil, being transported by rainwater into to the surface water. Nevertheless, concentrations of around 1.8 μg L^−1^ of mesotrione was found in streams next to crop fields [5]. This concentration was tested in our work and did not induced any changes in oxireductive enzymes in any species. Kreutz et al. [23] evaluated the LC_50_ of the neotropical fish *R. quelen* to many pesticides, including mesotrione. They found that mesotrione had a lethal concentration higher than other pesticides. While, the LC_50_ of mesotrione was 532 mg L^−1^, the LC50 of glyphosate, atrazine and tebuconazole were 7.3, 7.2, and 5.3 mg.L^−1^, respectively.

Although mesotrione is easily degradable, its metabolites can present toxic effects. The metabolites MNBA (4-methylsulfonyl-2-nitrobenzoic acid) and AMBA (2-amino-4- methylsulfonylbenzoic acid) are found together with the parental compound in both soil and water [1]. Bonnet et al. [6] compared the toxicity of atrazine with the mesotrione, they formulated commercial product (Callisto^®^), and its two major metabolites (MNBA and AMBA) in two nontarget microorganisms that are frequently used in ecotoxicological tests, the eukaryote *Tetrahymena pyriformis* and the prokaryote *Vibrio fischeri*. The results showed that the Callisto^®^ is more toxic to both species in comparison to atrazine, and about 80 times more toxic than mesotrione to *V. fischeri*. For the same microorganism, one of mesotrione metabolites, AMBA, was approximately 6 times more toxic than mesotrione itself. Ter Halle and Richard [36] showed that several photoproducts are formed in natural waters but only three were identified, MNBA is one of them. In a complete description of the biotransformation of mesotrione by a bacterial strain (*Bacillus* sp. 3B6) AMBA was identified as one of several other metabolites, all of which were present at relatively high concentrations [12], [13].

DNA damage can occur as a direct interaction of xenobiotics and its metabolites with the molecule or as a secondary consequence of oxidative stress. *O. niloticus* exposed to the two highest concentrations of mesotrione had DNA damage in all three organs tested in this study. *G. brasiliensis* also had significant increase in DNA damage in all organs, but in different concentrations of the pesticide. Many pesticides are known as genotoxic agents. Atrazine, for example, is known to increase the frequency of morphological nuclear abnormalities and DNA breakages in *O. niloticus* [39]. Ghisi and Cestari [16] showed increased DNA damage in the erythrocytes and hepatocytes of *Corydoras paleatus* exposed to sublethal concentrations of glyphosate. The same herbicide caused oxidative stress and genotoxic effects in *Astyanax* sp. [32]. Another triazinic herbicide, the 2-4-D was also induced oxidative stress and genotoxicity in *Oncorhynchus mykiss* [25]. Similar to other herbicides widely used in agriculture, mesotrione was capable of inducing genotoxicity in erythrocytes, hepatocytes, and gills cells of fish.

Although we did not observed increase in LPO levels in liver of *O. niloticus*, the GPx activity and GSH content was high in almost all treatments (7, 30 e 460 μg L^−1^). GSH works as an endobiotic molecule in the detoxification of various substrates [2], [37] and in the reduction of organic hydroperoxides (ROOH) via GST [3]. Moreover, GSH participates in the reduction of H_2_O_2_ in the reactions catalyzed by GPx, readily reacting with HO^−^ [3]. Therefore, the observed increase in GSH and GPx indicates that the defensive system against ROS was activated, protecting the fish from further damages to macromolecules, such as lipids and proteins, but not the DNA. The induction of ROS production by pesticides is widely studied, and it occurs in several species, including fish. In the work conducted by Mela et al. [26], *R. quelen* exposed to atrazine showed inhibited GPx, GST, GR, and CAT activity and reduction of GSH levels. This herbicide can be directly conjugated to GSH, decreasing their concentration and the activity of both GST and GPx, since they use GSH as substrate. Also in *R. quelen*, Glusczak et al. [17] demonstrated that occurred increases ROS levels can occur due to exposure to glyphosate. Activation of SOD and GPx also occurred in the gills of *Carassius auratus* exposed to the herbicide 2-4-D [4].

The activity of SOD was increased in groups 115 and 460 μg L^−1^ in *G. brasiliensis*. One of the first enzymes that act on the defense against ROS is superoxide dismutase (SOD), which catalyze the conversion of reactive superoxide anions to hydrogen peroxide (H_2_O_2_), which is subsequently detoxified by CAT and GPx [33]. Jin et al. [19] observed that SOD could be increased in liver of zebrafish exposed to herbicide atrazine in the concentration of 100 and 1000 μg L^−1^. Our results did not show increased activity of GPx in *G. brasiliensis*. However, there was a significant increase in the GST activity in the groups 7, 30 and 115 μg L^−1^. GST is present in the cytosol of many cells catalyzing the conjugation of GSH with many compounds and also acting in the elimination of oxygen radicals and reactive intermediates [27], which may being formed by the action of superoxide dismutase.

Exposure of animals to mesotrione triggered a defense response against ROS, as shown by a light increase of some systems including GPx activity and GSH content in *O. niloticus*, and GST and SOD activity in *G. brasiliensis*. Probably this response is enough to counteract ROS, because mesotrione only increased the LPO in the group 115 μg L^−1^ in *G. brasiliensis*, and any significant alteration in PCO was detected for both species. However, DNA damage occurred in both species (Fig. 1, Fig. 3). This suggests that ROS is not the main mechanism of the herbicide to induce genotoxicity.

Although it does not clarify the toxic mode of action of the mesotrione, our data is pioneer in testing the sublethal effects of mesotrione through the use of biomarkers in fish. For both studied species, this compound induced the responses of oxidoreductive enzymes, counteracting ROS, and was also genotoxic in concentrations lower to those applied on the field, but higher than that found in streams next to crop fields [5]. We encourage new studies and investigations that focus on the sublethal effects of such herbicide, its metabolites and the commercial formulated product.

Although it does not clarify the toxic mode of action of mesotrione, our data is pioneer in testing the sublethal effects of this herbicide through the use of biomarkers in fish. It is known that some herbicides, such as glyphosate, are able to induce a late oxidative damage to fish DNA [8], which can be repaired after a relatively short period by the activation of basic DNA repair pathways, *e.g.*, base and nucleotide excision repair photoreactivation or photoenzymatic repair, homologous recombination and non-homologous end-joining as well as the presence of poly (ADP-ribose) polymerases [22]. Mesotrione, in turn, despite being genotoxic in concentrations lower to those applied on the field but higher than the ones found in streams next to crop fields [5], induced the responses of oxidoreductive enzymes for both species analyzed in our work, which makes it uncertain whether this genotoxic effect is due to a late oxidative response, some other indirect or even a direct effect of the herbicide. Therefore, we encourage new studies and investigations that focus on the sublethal effects of mesotrione, its metabolites and the commercial formulated product in acute and chronic exposure.

## 5 Conclusion

Our data showed that the use of mesotrione in the crop fields must be closely observed. Despite its low concentration in nature, our results showed an increased DNA damage and oxidoreductive responses in fish exposed to such low concentrations. Thus, in order to have a broader environmental knowledge of the real toxic potential of mesotrione, it is necessary more studies with different species and different levels of contamination in order to better understand the toxicity of this compound, of its commercial formulation and of its metabolites in chronic and acute experiments.

## References

[1] Alferness P., Wiebe L. (2002). Determination of mesotrione residues and metabolites in crops, soil, and water by liquid chromatography with fluorescence detection. J. Agric. Food. Chem..

[2] Armstrong R.N. (1997). Structure, catalytic mechanism, and evolution of the glutathione transferases. Chem. Res. Toxicol..

[3] Arteel G.E., Sies H. (2001). The biochemistry of selenium and the glutathione system. Environ. Toxicol. Pharmacol..

[4] Atamaniuk T.M., Kubrak O.I., Storey K.B., Lushchak V.I. (2013). Oxidative stress as a mechanism for toxicity of 2,4-dichlorophenoxyacetic acid (2,4-D): studies with goldfish gills. Ecotoxicology.

[5] Barchanska H., Rusek M., Szatkowska A. (2012). New procedures for simultaneous determination of mesotrione and atrazine in water and soil. Comparison of the degradation processes of mesotrione and atrazine. Environ. Monit. Assess..

[6] Bonnet J.L., Bonnemoy F., Dusser M., Bohatier J. (2008). Toxicity assessment of the herbicides sulcotrione and mesotrione toward two reference environmental microorganisms: *Tetrahymena pyriformis* and *Vibrio fischeri*. Arch. Environ. Contam. Toxicol..

[7] Çavaş T., Ergene-Gözükara S. (2005). Micronucleus test in fish cells: a bioassay for in situ monitoring of genotoxic pollution in the marine environment. Environ. Mol. Mutagen..

[8] Çavas T., Könen S. (2007). Detection of cytogenetic and DNA damage in peripheral erythrocytes of goldfish (*Carassius auratus*) exposed to a glyphosate formulation using the micronucleus test and the comet assay. Mutagenesis.

[9] Cestari M.M., Lemos P.M.M., Ribeiro C.A.D.O., Costa J.R.M.A., Pelletier E., Ferraro M.V.M., Mantovani M.S., Fenocchio A.S. (2004). Genetic damage induced by trophic doses of lead in the neotropical fish *Hoplias malabaricus* (Characiformes, Erythrinidae) as revealed by the comet assay and chromosomal aberrations. Genet. Mol. Biol..

[10] Collins A.R., Dobson V.L., Dusinská M., Kennedy G., Stĕtina R. (1997). The comet assay: what can it really tell us?. Mutat. Res..

[11] Crouzet O., Batisson I., Besse-Hoggan P., Bonnemoy F., Bardot C., Poly F., Bohatier J., Mallet C. (2010). Response of soil microbial communities to the herbicide mesotrione: a dose-effect microcosm approach. Soil Biol. Biochem..

[12] Durand S., Amato P., Sancelme M., Delort A.M., Combourieu B., Besse-Hogan P. (2006). First isolation and characterization of a bacterial strain that biotransforms the herbicide mesotrione. Lett. Appl. Microbiol..

[13] Durand S., Légeret B., Martin A.S., Sancelme M., Delort A.M., Besse-Hoggan P., Combourieu B. (2006). Biotransformation of the triketone herbicide mesotrione by a *Bacillus* strain. Metabolite profiling using liquid hromatography/electrospray ionization quadrupole time-of-flight mass spectrometry. Rapid Commun. Mass Spectrom..

[14] Ferraro M.V.M., Fenocchio A.S., Mantovani M.S., Ribeiro C.D.O., Cestari M.M. (2004). Mutagenic effects of tributyltin and inorganic lead (Pb II) on the fish *H. malabaricus* as evaluated using the comet assay and the piscine micronucleus and chromosome aberration tests. Genet. Mol. Biol..

[15] Gao R., Yuan Z., Zhao Z., Gao X. (1998). Mechanism of pyrogallol autoxidation and determination of superoxide dismutase enzyme activity. Bioelectrochem. Bioenerg..

[16] Ghisi N.D.C., Cestari M.M. (2013). Genotoxic effects of the herbicide roundup^®^ in the fish *Corydoras paleatus* (Jenyns, 1842) after short-term, environmentally low concentration exposure. Environ. Monit. Assess..

[17] Glusczak L., Miron D.D.S., Moraes B.S., Simões R.R., Schetinger M.R.C., Morsch V.M., Loro V.L. (2007). Acute effects of glyphosate herbicide on metabolic and enzymatic parameters of silver catfish (*Rhamdia quelen*). Comp. Biochem. Physiol. C Toxicol. Pharmacol..

[18] Jiang Z.Y., Hunt J.V., Wolff S.P. (1992). Ferrous ion oxidation in the presence of xylenol orange for detection of lipid hydroperoxide in low density lipoprotein. Anal. Biochem..

[19] Jin Y., Zhang X., Shu L., Chen L., Sun L., Qian H., Liu W., Fu Z. (2010). Oxidative stress response and gene expression with atrazine exposure in adult female zebrafish (*Danio rerio*). Chemosphere.

[20] Johnson B.C., Young B.G. (2002). Influence of temperature and relative humidity on the activity of mesotrione. Weed Sci..

[21] Keen J.H., Habig W.H., Jakoby W.B. (1976). Mechanism for the several activities of the glutathione S-transferases. J. Biol. Chem..

[22] Kienzler A., Bony S., Devaux A. (2013). DNA repair activity in fish and interest in ecotoxicology: a review. Aquat. Toxicol..

[23] Kreutz L.C., Barcellos L.J.G., Silva T.O., Anziliero D., Martins D., Lorenson M., Marteninghe A., da Silva L.B. (2008). Acute toxicity test of agricultural pesticides on silver catfish (*Rhamdia quelen*) fingerlings. Cienc. Rural.

[24] Levine R.L., Williams J.A., Stadman E.R., Shacter E. (1994). Carbonyl assays for determination of oxidatively modified proteins. Method Enzymol..

[25] Martínez-Tabche L., Madrigal-Bujaidar E., Negrete T. (2004). Genotoxicity and lipoperoxidation produced by paraquat and 2,4-dichlorophenoxyacetic acid in the gills of rainbow trout (*Oncorhynchus mikiss*). Bull. Environ. Contam. Toxicol..

[26] Mela M., Guiloski I.C., Doria H.B., Randi M.A.F., De Oliveira Ribeiro C.A., Pereira L., Maraschi A.C., Prodocimo V., Freire C.A., Silva de Assis H.C. (2013). Effects of the herbicide atrazine in neotropical catfish (*Rhamdia quelen*). Ecotoxicol. Environ Saf..

[27] Melgar Riol M.J., Nóvoa Valiñas M.C., García Fernández M.A., Pérez López M. (2001). Glutathione S-transferases from rainbow trout liver and freshly isolated hepatocytes: purification and characterization. Comp. Biochem. Physiol. C Toxicol. Pharmacol..

[28] Osório F.H.T., Silva L.F.O., Piancini L.D.S., Azevedo A.C.B., Liebel S., Yamamoto F.Y., Philippi V.P., Oliveira M.L.S., Ortolani-Machado C.F., Filipak Neto F., Cestari M.M., da Silva de Assis H.C., de Oliveira Ribeiro C.A. (2013). Water quality assessment of the Tubarão River through chemical analysis and biomarkers in the neotropical fish *Geophagus brasiliensis*. Environ. Sci. Pollut. Res. Int..

[29] Paglia D.E., Valentine W.N. (1967). Studies on the quantitative and qualitative characterization of erythrocyte glutathione peroxidase. J. Lab. Clin. Med..

[31] Ramsdorf W., Guimaraes F., Ferraro M., Gabardo J., Trindade E., Cestari M. (2009). Establishment of experimental conditions for preserving samples of fish blood for analysis with both comet assay and flow cytometry. Mutat. Res.

[32] Rossi S.C., Dreyer da Silva M., Piancini L.D.S., Oliveira Ribeiro C.A., Cestari M.M., Silva de Assis H.C. (2011). Sublethal effects of waterborne herbicides in tropical freshwater fish. Bull. Environ. Contam. Toxicol..

[33] Santos T.G., Martinez C.B.R. (2012). Atrazine promotes biochemical changes and DNA damage in a neotropical fish species. Chemosphere.

[34] Sedlak J., Lindsay R.H. (1968). Estimation of total, protein-bound, and nonprotein sulfhydryl groups in tissue with Ellman's reagent. Anal. Biochem..

[35] Speit G., Hartmann A. (1999). The comet assay (single-cell gel test). A sensitive genotoxicity test for the detection of DNA damage and repair. Methods Mol. Biol..

[36] Ter Halle A., Richard C. (2006). Simulated solar irradiation of mesotrione in natural waters. Environ. Sci. Technol..

[37] Van Bladeren P.J. (2000). Glutathione conjugation as a bioactivation reaction. Chem. Biol. Interact..

[38] Van der Oost R., Beyer J., Vermeulen N. (2003). Fish bioaccumulation and biomarkers in environmental risk assessment: a review. Environ. Toxicol. Pharmacol..

[39] de Ventura B.C., de Angelis D.D.F., Marin-Morales M.A. (2008). Mutagenic and genotoxic effects of the atrazine herbicide in *Oreochromis niloticus* (Perciformes, Cichlidae) detected by the micronuclei test and the comet assay. Pestic. Biochem. Physiol..

